# Off-licence use of clozapine in patients with emotionally unstable personality disorder: a case series analysis

**DOI:** 10.1192/bjo.2021.339

**Published:** 2021-06-18

**Authors:** Declan Hyland, Alex Walmsley, Victoria Simpson

**Affiliations:** 1 Consultant Psychiatrist, Clock View Hospital, Liverpool, Mersey Care NHS Foundation Trust; 2 5th year medical undergraduate, University of Liverpool

## Abstract

**Objective:**

This retrospective case series followed emotionally unstable personality disorder (EUPD) patients following initiation of clozapine on an off-licence basis, aiming to examine tolerance by determining side effect prevalence and treatment cessation frequency, as well as examining efficacy, by investigating number of hospital re-admissions and symptom control.

**Case report:**

This case series captured the experiences of 11 EUPD patients under the care of Mersey Care NHS Foundation Trust, all of whom had, at some time in the past five years, been initiated on clozapine. All patients were white British females, with a median age of 31. The median daily dose of clozapine was 300 mg. Most patients had significant psychiatric comorbidities, as well as illicit substance and / or alcohol misuse.

Whilst prescribed clozapine, patients were only admitted to hospital once on average and this was commonly for clozapine re-titration. Whilst in hospital, rates of self-harm were low, but ligaturing and suicide attempts showed higher prevalence. Patients still demonstrated self-harming behaviour out of hospital leading to A and E presentations. In the community, contacts with the police were minimal, with only two patients undergoing Section 136 assessments or arrests.

All patients reported side effects from clozapine - usually hypersalivation, over-sedation and constipation. All 11 patients experienced sinus tachycardia. Eight patients temporarily ceased taking clozapine at some point. In three patients, discontinuation of clozapine was as a result of intolerable side effects. Three patients experienced neutropenia, which subsequently resolved. Only two patients had a body mass index within healthy range.

**Discussion:**

Despite patients reporting clozapine to provide symptomatic benefit for their EUPD, and improved their engagement with mental health services, prevalence of self-harm and of A and E presentations remained high, indicating the importance of community support and concomitant psychotherapeutic treatment. Patients with more robust community support showed greater adherence to clozapine.

High prevalence of side effects and obesity in these patients, in addition to risk of developing neutropenia, highlights the importance of rigorous monitoring after initiating clozapine. It is reassuring that, despite development of neutropenia in some patients, this recovered quickly, and clozapine treatment could resume.

**Conclusion:**

Clozapine may be an effective pharmacological treatment for enabling EUPD patients to engage more therapeutically with services. Clozapine may be of greater benefit to those with more stable, less chaotic lives. Although diminished, patients still show self-harming behaviour and need for A and E admissions and re-hospitalisation. Side effects of clozapine are common and regular monitoring is required.

